# Imaging for molecular and pathological subtyping of hepatocellular carcinoma—a critical appraisal and future directions

**DOI:** 10.1007/s00330-025-12075-1

**Published:** 2025-10-30

**Authors:** Xinyuan Jia, Hanyu Jiang, Zheng Ye, Hong Wei, Jie Chen, Yali Qu, Claude B. Sirlin, Bin Song, Yanshu Wang

**Affiliations:** 1 Department of Radiology, Functional and Molecular Imaging Key Laboratory of Sichuan Province, West China Hospital, Sichuan University, Chengdu, China; 2 Liver Imaging Group, Department of Radiology, University of California-San Diego, San Diego, CA USA; 3 Department of Radiology, Sanya People’s Hospital, Sanya, China

**Keywords:** Hepatocellular carcinoma, Computed tomography, Magnetic resonance imaging, Prognosis, Molecular and pathological classification

## Abstract

**Objectives:**

Hepatocellular carcinoma (HCC) is characterized by distinct molecular and pathological subtypes, each with unique prognostic implications. This review aims to synthesize the imaging features associated with these HCC subtypes and discuss their potential to guide therapeutic decision-making.

**Materials and methods:**

We searched PubMed and Embase for articles published from September 2004 to December 2024. The search strategy combined terms for imaging modalities (“CT,” “MRI”), the primary disease (“hepatocellular carcinoma”), and various molecular and pathological subtypes (e.g., “macrotrabecular-massive,” “steatohepatitic,” “CK19,” and “*CTNNB1*”).

**Results:**

HCC is a biologically heterogeneous malignancy with varied prognosis and sensitivity to treatment. Assessment of its molecular and pathological subtypes relies on invasive histopathological examination, which is subject to sampling errors and often unavailable prior to treatment selection. A growing body of evidence suggests that radiologic features aid in the non-invasive classification of HCC subtypes, thereby informing individualized therapy. Given the substantial overlap between molecular, pathological, and imaging features, this review hypothesize that a comprehensive phenotyping system integrating all these information could significantly enhance personalized prognostication and treatment strategies.

**Conclusion:**

Radiologic imaging features not only provide valuable information for identifying molecular and pathological subtypes of HCC but also serve as practical tools to guide individualized therapeutic decision-making.

**Key Points:**

***Question*** *Can CT and MRI reliably infer the molecular classification and pathological subtypes that drive prognosis in HCC*?

***Findings*** *Several imaging features have been found to reflect underlying molecular and pathological subtypes, but they do not demonstrate a one-to-one correlation*.

***Clinical relevance*** *An integrated classification system incorporating clinical, imaging, pathological, and molecular data may help mitigate the limitations of histologic and molecular analyses and facilitate individualized prognostication*.

## Introduction

Hepatocellular carcinoma (HCC) is a biologically heterogeneous malignancy [1, 2]. Such heterogeneity complicates management by influencing the risks of recurrence, metastasis, and therapeutic response [3, 4]. Several classifications have been proposed to stratify HCC into distinct subtypes with varied molecular and pathological characteristics and prognoses. However, assessment of HCC subtypes, whether molecular or pathological, requires invasive tissue sampling, which is often unavailable at the time of therapeutic decision-making and subject to sampling errors. Accumulating evidence suggests that key molecular and pathological characteristics may be inferred from multiphase computed tomography (CT) and multiparametric magnetic resonance imaging (MRI) (e.g., qualitative, quantitative, and radiomic features) [5–8]. Thus, understanding the correlations between imaging features, molecular characteristics, and pathological subtypes may improve noninvasive outcome prognostication and treatment responsiveness prediction, thereby informing individualized HCC management.

This review summarizes the imaging features associated with molecular and pathological subtypes of HCC, with attention given to the current understanding of these subtypes’ pathological and molecular bases and relevance to clinical practice.

## Molecular and pathological HCC classifications

### Molecular classification

Molecular heterogeneity drives biological heterogeneity. According to gene expression profiles, major molecular drivers and altered signaling pathways, HCCs could be broadly divided into the proliferative and non-proliferative classes, each with various subclasses [9–14]. Generally, the proliferative class is characterized by active cell cycle progression, uncontrolled cell division, proliferative signaling pathways, and genomic chaos with chromosomal instability and aberrant epigenetic alterations. Notably, proliferative tumors may exhibit some but not all of these abnormalities; the key concept is that the global molecular profile is one of proliferation. The non-proliferative class is characterized by retained hepatocyte-like features with altered cellular metabolism or activation of inflammatory pathways but without uncontrolled proliferation. Given tumor heterogeneity, HCCs may harbor both proliferative and non-proliferative components at the lesion or patient levels, but this has not yet been investigated.

The proliferative class accounts for approximately 50% of HCC, with the non-proliferative class comprising the remaining portion [2, 9]. Proliferative class is more frequently observed in patients with chronic hepatic B virus (HBV) infection and has been associated with a more aggressive phenotype (e.g., poorer differentiation and greater propensity for vascular invasion, satellite nodules, and metastasis), higher serum alpha-fetoprotein (AFP) and des-gamma-carboxy prothrombin (DCP) levels, and poorer clinical outcomes, even after adjusting for tumor size and stage [15, 16]. Proliferative HCCs demonstrate enrichment of proliferation-related signaling pathways, such as PI3K-AKT-mTOR, RAS-MAPK, and MET cascades, as well as high chromosomal instability, global DNA hypomethylation, and frequent *TP53* inactivation with *FGF19* or *CCND1* amplification. Further subclassification of the proliferative class reveals (1) a “Wnt-TGFβ subclass”, characterized by activation of both TGFβ and Wnt pathways [17] and (2) a “progenitor subclass” with expression of epithelial cell adhesion molecule (EpCAM) or cytokeratin 19 (CK19), and upregulation of Myc and Akt protein kinase [16, 18].

Conversely, the non-proliferative class predominates in alcohol-related steatotic liver disease (ALD), metabolic dysfunction-associated steatohepatitis (MASH), and hepatitis C virus (HCV)-related HCCs, and has been associated with a less aggressive phenotype, lower AFP levels, and better clinical outcomes. Molecularly, these tumors are characterized by higher chromosomal stability, extensive promoter hypermethylation, and more frequent mutations in *CTNNB1* and *TERT* promoters, resulting in altered metabolic regulation and cancer growth with relatively low proliferative activity [10, 19]. Why ALD, MASH, and chronic HCV predispose to non-proliferative HCC is not yet understood, but vascular, metabolic, immune, and inflammatory factors may play a role. Importantly, the clinical associations mentioned above (e.g., of proliferative HCC with chronic HBV, higher AFP and DCP; of non-proliferative HCC with other etiologies of liver disease and lower AFP) are not strong enough to enable noninvasive classification of tumors based on this information alone.

Emerging evidence suggests that molecular alterations in HCC may have prognostic and therapeutic implications. For instance, *CTNNB1* mutations have been reported to drive tumor immune exclusion, and patients with *CTNNB1* mutations are often considered to be resistant to immunotherapy [20]. However, considering the complexity and cost of genetic analyses, pathological classification (detailed below) may serve as a practical surrogate.

### Pathological subtypes

In parallel to molecular heterogeneity, HCC also exhibits substantial morphological heterogeneity at the pathological level. The 2019 WHO classification (5th edition) classifies up to 65% of HCCs as conventional HCCs (also known as “not otherwise specified” subtype), lacking distinctive morphological characteristics [21]. The remaining 35% are classified into one of the following eight subtypes: macrotrabecular-massive (MTM), steatohepatitic (SH), clear-cell, scirrhous, chromophobe, fibrolamellar, neutrophil-rich, and lymphocyte-rich HCC [22]. Below are the pathological characteristics of common histological subtypes; those with an incidence < 5% are detailed in Supplementary Material 1.

**Fig. 1:**
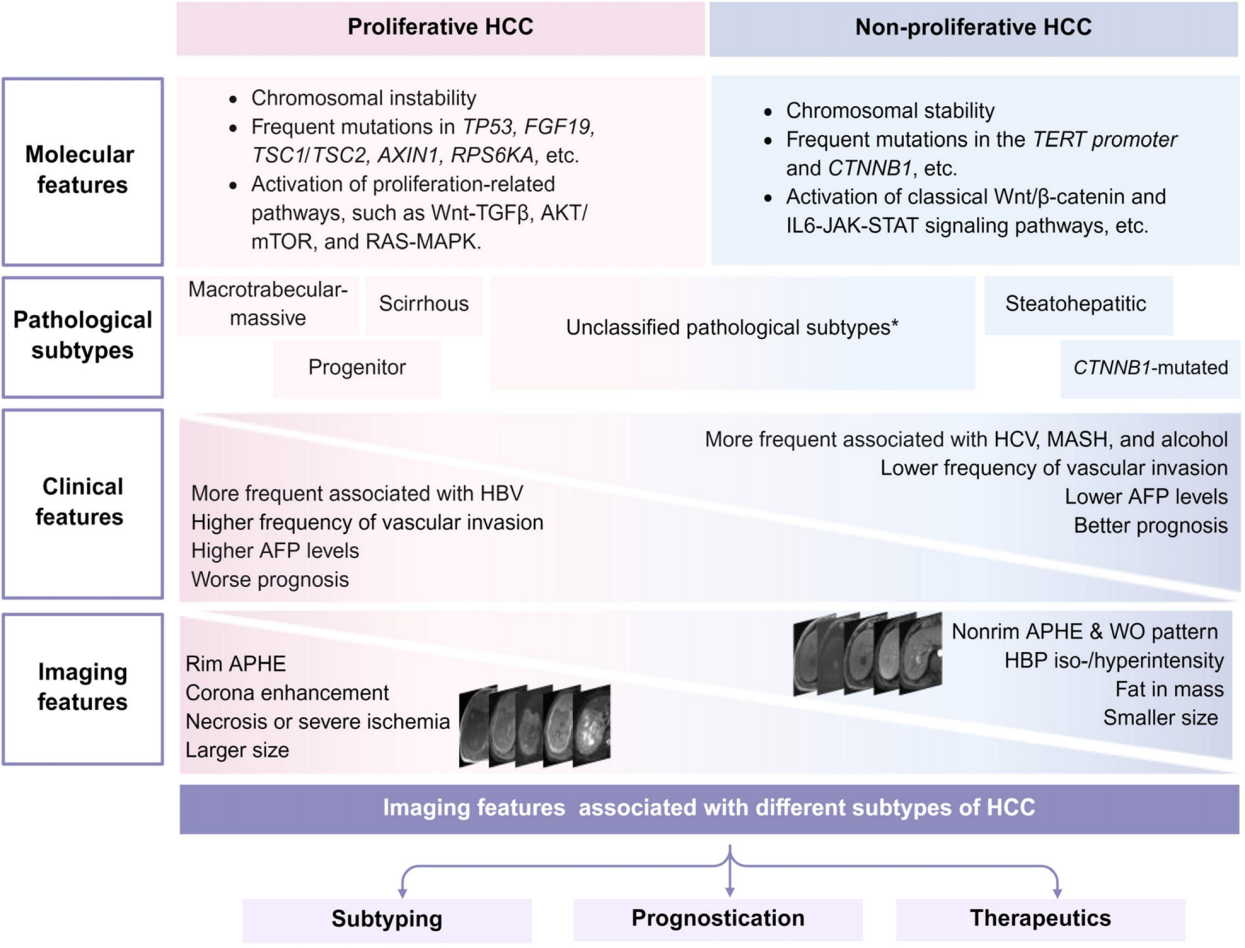
An illustration highlighting the predictive value of imaging features in identifying the integrated molecular and pathological classification of HCC. *Unclassified subtypes include pathological variants with uncertain molecular classification according to current evidence, such as not otherwise specified (NOS), neutrophil-rich, clear cell, chromophobe, fibrolamellar, and lymphocyte-rich HCCs, etc. HCC, hepatocellular carcinoma; *TP53,* tumor protein p53; *FGF19,* fibroblast growth factor 19; *TSC*, tuberous sclerosis; *TERT,* telomerase reverse transcriptase; MTM, macrotrabecular-massive; AFP, alpha-fetoprotein; HBV, hepatitis B virus; HCV, hepatitis C virus; MASH, metabolic dysfunction-associated steatohepatitis; APHE, arterial phase hyperenhancement; WO, “Washout” or washout appearance

The pinpoint relationship between molecular and pathological classifications remains inadequately established. However, the phenotype of HCC is unlikely to be random, but rather reflects genomic traits. Specifically, the MTM and progenitor subtypes are generally classified within the proliferative class [9], whereas the steatohepatitic and *CTNNB1*-mutated subtypes are typically classified within the non-proliferative class (Fig. 1) [11]. To date, molecular investigations for neutrophil-rich HCCs, clear-cell HCCs, and lymphocyte-rich HCCs remain limited. Some studies have classified neutrophil-rich HCCs as proliferative and clear-cell and lymphocyte-rich HCCs as non-proliferative based on their behavioral patterns and prognosis, although this classification remains controversial [23, 24].

#### Proliferative class

**(1) MTM-HCC**

MTM-HCC has been observed in approximately 5% of HCC and characterized by a prominent macrotrabecular growth pattern (HCC cords > 6 cells in thickness) in ≥ 50% of the tumor [9, 16]. Molecularly, MTM-HCC is characterized by frequent *TP53* mutation and *FGF19* amplification, and is generally regarded within the proliferative class. Pathologically, this subtype has been associated with larger size and aggressiveness, including higher tumor grade and more frequent intratumoral necrosis, microvascular invasion (MVI), vessels encapsulating tumor clusters, and satellite nodules [15, 25]. Clinically, MTM-HCC has been linked to poorer prognosis with increased metastatic spread and macrovascular invasion, even after adjusting for initial size, grade, and stage [15, 16]. Recent studies have proposed that patients with MTM-HCC may benefit from a combination of anti-CMTM6 and anti-programmed death-ligand 1 (anti-PD-L1) therapy, given the role of CMTM6 in stabilizing PD-L1 expression and enhancing immune evasion by preventing its degradation [26, 27].

**(2) Progenitor HCC**

The reported proportion of progenitor subtype ranges from 10% to 35% of HCC and is characterized by > 5% hepatic progenitor cell expression (e.g., CK19 or EpCAM) on immunohistochemistry [28, 29]. Compared to non-progenitor HCCs, these tumors are characterized by larger size, infiltrative growth with reduced capsule formation (potentially due to enhanced cytoskeletal dynamics and cellular motility), and frequent vascular invasion. Molecularly, they are characterized by expression of epithelial-mesenchymal transition-related proteins [28]. The aggressive biological behavior results in poorer prognosis, higher metastatic potential, and worse disease-free survival after curative-intent surgery, even after adjusting for size and stage [28–31]. Furthermore, they are more resistant to sorafenib and transarterial chemoembolization (TACE), while novel therapies targeting SALL4, a key mediator of HCC stemness, may offer therapeutic benefits [30, 32].

#### Non-proliferative class

**(1)** **Steatohepatitic hepatocellular carcinoma** **(SH-HCC)**

SH-HCC represents 5–20% of HCC and is defined by ≥ 5% tumor steatohepatitis (i.e., steatosis, ballooning, inflammation, and pericellular fibrosis) [33]. The key molecular features include IL-6/JAK/STAT activation and less frequent *CTNNB1*, *TERT*, and *TP53* mutations. Pathologically, these tumors exhibit a less aggressive phenotype with infrequent MVI and metastases. Clinically, SH-HCC has been more frequently observed in patients with ALD or MASH and may exhibit increased resistance to immunotherapies due to an immune-suppressive tumor microenvironment driven by exhausted CD8^+^ PD1^+^T cells [34, 35].

**(2)** ***CTNNB1-*mutated HCC**

The *CTNNB1*-mutated HCC is generally well-differentiated, characterized by trabecular and pseudo-glandular architectural patterns, intratumoral cholestasis, and lack of immune infiltrates. *CTNNB1-*mutated HCC accounts for 30–40% of cases and is characterized by canonical WNT/β-catenin signaling activation. Given the limited sensitivity of β-catenin detection, studies also utilize positive expression (> 5%) of β-catenin or glutamine synthetase (GS, a downstream protein regulated by β-catenin) as the diagnostic criteria for *CTNNB1-*mutated HCC [7, 36]. Pathologically, *CTNNB1-*mutated HCC is frequently associated with large tumor size (> 5 cm), well-differentiated histology, cholestasis, and microtrabecular/pseudoglandular patterns [9]. Studies have reported an increased resistance to immune checkpoint inhibitors (ICIs) due to decreased T-cell infiltration within the tumor. Notably, promising studies have shown that targeting the Wnt/β-catenin pathway with PORCN inhibitors (i.e., ETC-159) or the mTOR inhibitors (i.e., Rapamycin), may have therapeutic benefits for this subtype. Despite its immune-cold phenotype, *CTNNB1*-mutated HCC may be associated with more favorable clinical outcomes compared to conventional HCC [20, 24, 37].

## Imaging features of HCC molecular and pathological subtypes

**Fig. 2:**
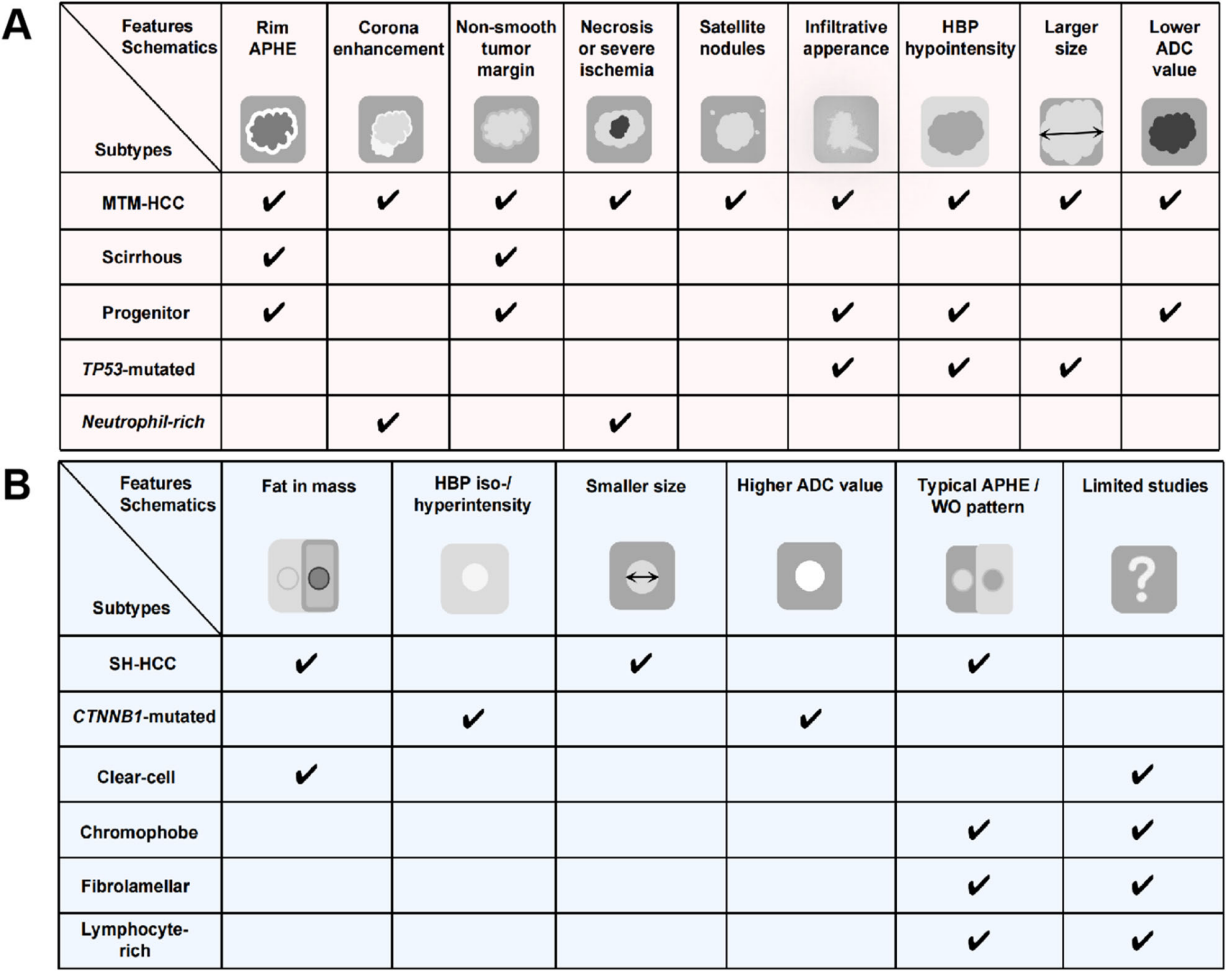
Schematic representation of common radiologic features and their correlations with molecular and pathological subtypes of HCC. **A** Radiologic features of the Proliferative class. **B** Radiologic features of the Non-Proliferative class. HCC, hepatocellular carcinoma; APHE, arterial phase hyperenhancement; HBP, hepatobiliary phase; ADC, apparent diffusion coefficient; MTM, macrotrabecular-massive; CK-19, cytokeratin-19; TP53, tumor protein p53; WO, WO “Washout” or washout appearance.

Radiologist-defined “semantic” features and computer-derived “radiomic” features on CT and MRI enable noninvasive profiling of HCC molecular and pathological subtypes (Fig. 2) [38–40]. Although radiotracers were also reported to be useful, this review focuses on CT and MRI features (Tables 1 and  2).

**Table 1:** Imaging features associated with proliferative HCC and their predictive or diagnostic performance

Subtypes	Imaging features		MRI images	Definitions	Frequencies	ORs/diagnostic metrics (sensitivities, specificities, AUCs)^†^
Proliferative class	Qualitative	Rim APHE*		Spatially defined subtype of arterial phase hyperenhancement in which arterial phase enhancement is most pronounced in the tumor periphery [23, 38, 70].	42.2–61.9%	OR = 2.79–12.3 Sensitivity = 61.9% Specificity = 88.8%
		Mosaic architecture*		Presence of any combination of internal nodules, compartments, or septations, within a solid or mostly solid mass [38].	28%	OR = 3.3
		Satellite lesion		Presence of distinct, unequivocal HCC(s) that are smaller than 1 cm and within 2 cm of the main tumor [38].	8.70%	OR = 3.2
		Lobulated shape		A tumor with a lobulated appearance [38].	30.80%	OR = 2.1
		Corona enhancement*		Peritumoral enhancement in the late arterial phase or early portal venous phase. The enhancement is contiguous with and surrounds all or part of the tumor [70].	31.0–57.4%	OR = 3.37
		Infiltrative appearance*		A non-circumscribed tumor margin (indistinct transition) covering > 50% of the tumor circumference may indicate permeative growth [70].	18.0-34.4%	OR = 7.82
		Intratumoral artery		Presence of discrete arteries within the tumor on AP [70].	31.0–55.7%	OR = 4.71
		Substantial hypoenhancing component		The enhancement of over 50% of the tumor area is considerably lower than that of the liver parenchyma in AP [70].	41.4–54.1%	OR = 2.68
	Quantitative	Radiomics and deep learning		NA	NA	AUC = 0.80–0.99
MTM-HCC	Qualitative	Intratumoral necrosis (Necrosis or severe ischemia*, substantial necrosis, hypovascular component on AP)		Presence of nonenhancing area in a solid mass, not attributable to a cystic component, prior treatment, or intralesional hemorrhage [6, 41, 45, 47]; OR presence of a central area of high-signal intensity on T2WI without enhancement and involving at least 20% of the tumor area at the level of the largest cross-sectional diameter [44, 57]; OR the presence of a hypovascular component during AP, occupying at least 20% of the total tumor volume [42].	21.4–93.0%	OR = 3.32–32.0 Sensitivity = 65–88% Specificity = 57–93%
		Intratumoral hemorrhage		Presence of a hyperattenuated area with a relatively lower attenuation compared with calcification on the nonenhanced images [45].	15.0%	OR = 5.4
		Rim APHE*		Spatially defined subtype of arterial phase hyperenhancement in which arterial phase enhancement is most pronounced in the tumor periphery [41, 47].	~15%	OR = 2.63 Sensitivity = 16% Specificity = 92%
		Intratumoral artery		Presence of discrete arteries within the tumor on AP [42, 47].	45.0–67.0%	OR = 2.58 Sensitivity = 45-58% Specificity = 76–78%
		Corona enhancement*		Peritumoral enhancement in the late arterial phase or early portal venous phase. The enhancement is contiguous with and surrounds all or part of the tumor [42, 47].	51.0%	OR = 2.55 Sensitivity = 26–51% Specificity = 78–88%
		Non-smooth tumor margin		The tumor margin is irregular and/or has areas of bulging, nodular projection, or infiltration into adjacent tissues at the tumor periphery in any imaging plane [42, 47].	67–76%	Sensitivity = 67% Specificity = 65%
		Tumor in vein*		Unequivocal presence of enhancing soft tissue in the vein, regardless of the presence of parenchymal mass [42].	14.3–35%	OR = 2.35 Sensitivity = 21% Specificity = 89%
		Infiltrative appearance*		A non-circumscribed tumor margin (indistinct transition) covering > 50% of the tumor circumference may indicate permeative growth [41].	~25%	NA
		Peritumoral HBP hypointensity		Presence of irregular, wedge-shaped, or flame-like hypointense area of liver parenchyma located outside of the tumor margin in the hepatobiliary phase [47].	NA	OR = 2.21 Sensitivity = 44% Specificity = 71%
	Quantitative	Tumor size* (larger)		The outer-edge-to-outer-edge dimension of a tumor is larger than that of non-MTM HCC [6, 44].	NA	NA
		Low ADC value		ADC value of the tumor in whole or in part is lower than the liver [6].	NA	OR = 3.05 Sensitivity = 59% Specificity = 68%
		Radiomics and deep learning		NA	NA	AUC = 0.74–0.91
Progenitor HCC	Qualitative	Non-smooth tumor margin		The tumor margin is irregular and/or has areas of bulging, nodular projection, or infiltration into adjacent tissues at the tumor periphery in any imaging plane [31, 50, 51].	73.7–81.6%	OR = 3.05–6.66 Sensitivity = 74.0–81.6% Specificity = 54.9–70%
		Rim APHE*		Spatially defined subtype of arterial phase hyperenhancement in which arterial phase enhancement is most pronounced in the tumor periphery [31].	36.80%	OR = 8.71 Sensitivity = 36.8% Specificity = 70.1%
		Targetoid appearance*		Including targetoid restriction and targetoid TP or HBP appearance, as defined in LI-RADS v2018 [50].	42.1%	OR = 26.17 Sensitivity = 42% Specificity = 97%
		Mosaic architecture* (absence)		Absence of any combination of internal nodules, compartments, or septations, within a solid or mostly solid mass [50].	21.0%	OR = 15.7 Sensitivity = 21% Specificity = 92%
		Progressive or persistent dynamic enhancement pattern		Either a gradual increase in solid tumor enhancement that peaks in the delayed phase or a consistent intensity throughout the arterial and venous phases [51].	25.0%	NA
	Quantitative	Low tumor-to-liver ADC ratio		Tumor-to-liver ADC ratio ≤ 0.82 [31]	NA	OR = 13.28 Sensitivity = 36.8% Specificity = 93.6%
		Low tumor-to-liver SI on HBP		Tumor-to-liver SI ratio on HBP ≤ 0.52 [31]	NA	OR = 3.12 Sensitivity = 92.1% Specificity = 59.3%
		Radiomics and deep learning		NA	NA	AUC = 0.73–0.85

* Represents the radiologic feature is included in LI-RADS v2018, otherwise not

^†^ The presentation of ORs or diagnostic metrics (sensitivities, specificities, AUCs) is based on the availability of data provided in the original studies

*OR* odds ratio, *AUC* area under the curve, *HCC* hepatocellular carcinoma, *MTM* macrotrabecular-massive, *AP* arterial phase, *APHE* arterial phase hyperenhancement, *HBP* hepatobiliary phase, *ADC* apparent diffusion coefficient, *T2WI* T2-weighted imaging, *T1WI* T1-weighted imaging, *SI* signal intensity, *TP* transitional phase, *NA* not available, *LI-RADS* liver imaging reporting and data system

**Table 2:** Imaging features associated with non-proliferative HCC and their predictive or diagnostic performance

Subtypes	Imaging features		MRI images	Definitions	Frequencies	ORs/AUCs^†^
SH-HCC	Qualitative	Fat in mass*		Excess fat within a mass, in whole or in part, relative to the adjacent liver [41, 57].	26.7–66.7%	OR = 15.07
	Quantitative	Tumor size* (smaller)		The outer-edge-to-outer-edge dimension of a tumor is smaller than that of non-SH HCC [41, 57].	NA	NA
*CTNNB1*-mutated	Qualitative	HBP iso-/hyperintensity		Signal intensity of the tumor in the hepatobiliary phase (nearly) identical to or higher than that of the liver, in whole or in part [7].	NA	NA
	Quantitative	Tumor size* (larger)		The outer-edge-to-outer-edge dimension of a tumor is larger than that of HCCs without CTNNB1 mutations [16].	NA	OR = 2.14
		Higher ADC value	NA	Calculated as: ADC = Log [SI_1_/ SI_2_] / [b_2_–b_1_], where SI_1_ and SI_2_ are the SIs of ROI_1 and_ ROI_2_, with b_1_ = 0 s/mm² and b_2_ = 800 s/mm² [7].	NA	NA
		Radiomics and deep learning	NA	NA	NA	AUC = 0.70–0.88 [66, 67]

* Represents the radiologic feature is included in LI-RADS v2018, otherwise not

^†^ The presentation of ORs or AUCs is based on the availability of data provided in the original studies

*SH* steatohepatitic, *OR* odds ratio, *AUC* area under the curve, *HCC* hepatocellular carcinoma, *HBP* hepatobiliary phase, *ADC* apparent diffusion coefficient, *SI* signal intensity, *ROI* region of interest, *NA* not available, *LI-RADS* liver imaging reporting and data system

### Proliferative class

#### MTM-HCC

On CT/MRI, MTM-HCC has been associated with larger tumor size, higher frequency of substantial necrosis and hypovascular component on arterial phase (AP), tumor in vein and Liver Imaging Reporting and Data System M (LI-RADS M) features (e.g., rim arterial phase hyperenhancement [APHE]) compared with non-MTM-HCC [41, 42].

Specifically, Rhee et al reported that irregular rim APHE on gadoxetic acid-enhanced MRI (EOB-MRI) were more frequently observed in tumors with macrotrabecular patterns in a single-center retrospective study [5]. In a subsequent multicenter study, the same team further found that the hypovascular component ≥ 20% on AP could be used to diagnose MTM-HCCs with a negative predictive value of over 90%. When the hypovascular component exceeded 50%, along with ≥ 2 ancillary findings (i.e., intratumoral artery, peritumoral enhancement on AP, and non-smooth tumor margin), the diagnostic specificity increased to 96% [42]. On CT, Rhee and colleagues found this criterion to be as effective as on EOB-MRI for the diagnosis of the MTM subtype and for prognostication, although the included patients overlapped completely with their previous work [43]. Similarly, Mulé et al reported a specificity of 93% for substantial necrosis (the absence of enhancement combined with T2 hyperintensity in ≥ 20% of the tumor’s maximal diameter) for identifying MTM-HCCs [44]. Despite promising results reported thus far, defining the hypovascular or necrotic component ratio may be challenging and increase inter-reader variability [41, 44].

**Fig. 3:**
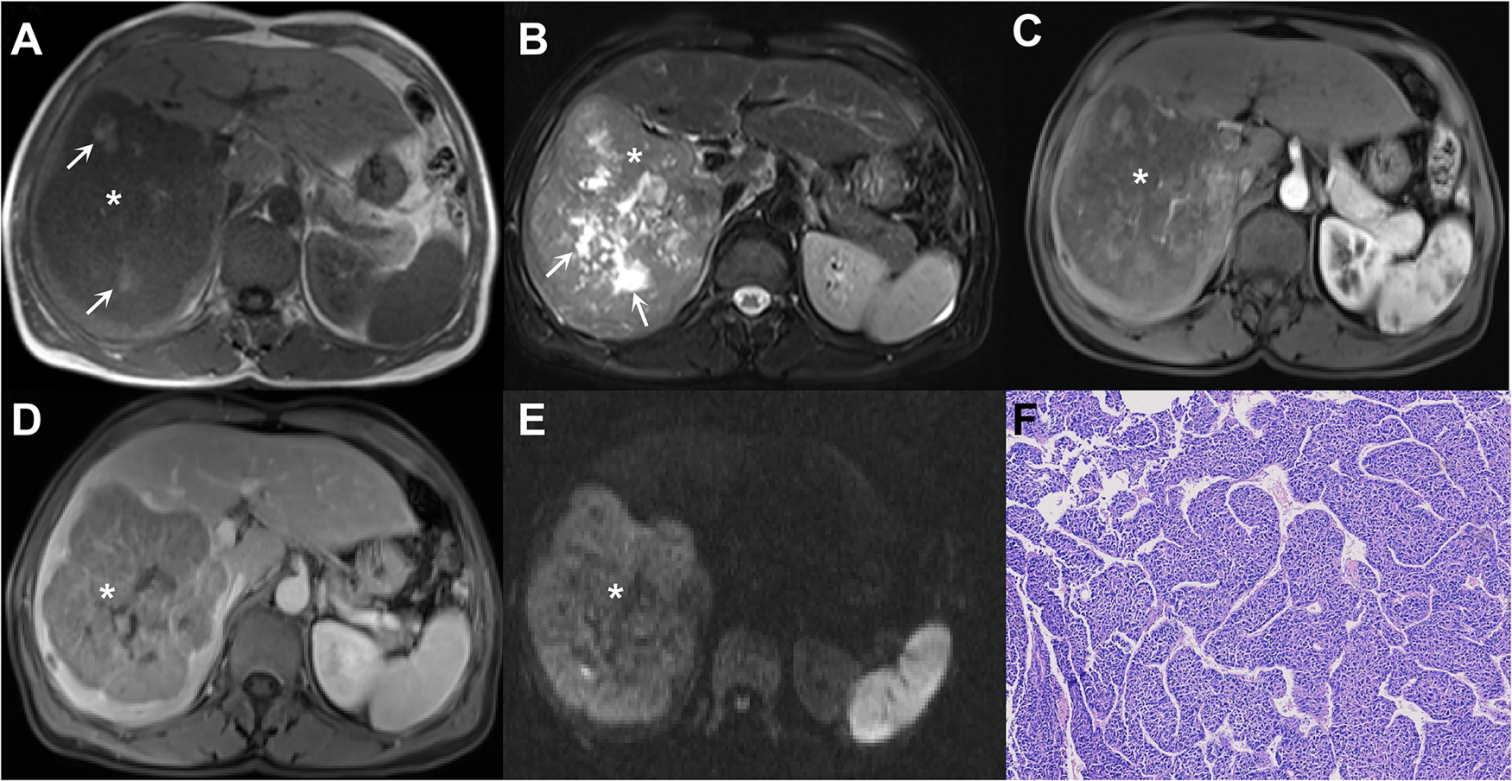
Imaging of a 62-year-old male with chronic hepatitis B virus infection and macrotrabecular-massive HCC (*, **A**–**E**). The lesion shows focal hyperintensity on the T1-weighted image (arrow in **A**), suggestive of intratumoral hemorrhage, and hyperintensity on the T2-weighted image (arrow in **B**), indicative of intratumoral necrosis. The lesion also demonstrates non-rim APHE on arterial phase image (**C**), nonperipheral washout on portal venous phase image (**D**), and marked diffusion restriction on diffusion weighted image (**E**). Hematoxylin-eosin staining under light microscopy reveals a macrotrabecular pattern (**F**). The patient underwent surgical resection and died 6 months postoperatively

Integrating clinical factors further enhances the noninvasive diagnostic performance for MTM-HCC [6, 45, 46]. For example, Feng et al proposed an ANH score (AFP, necrosis, and hemorrhage) based on CT for diagnosing MTM-HCCs, achieving an area under the curve (AUC) of 0.73 in the external validation cohort. Interestingly, substantial necrosis was also related to the vascular endothelial-to-tumor cell transition pattern. Similarly, Chen et al developed a nomogram incorporating high platelet count (≥ 163.5 × 103/µL), low tumor-to-liver apparent diffusion coefficient (ADC) ratio (≤ 1.05), and necrosis or severe ischemia, which achieved an AUC of 0.81 in the validation dataset. The aforementioned conclusions were not entirely consistent, possibly due to differences in study populations and surgical resection indications. Moreover, some of the associations (e.g., high platelet count) are not well understood. Nonetheless, they uniformly highlighted the utility of intratumoral necrosis in diagnosing MTM-HCCs. This finding was further verified in a meta-analysis, which showed the highest pooled odds ratio of intratumoral necrosis (4.16) for the diagnosis of MTM-HCC (Fig. 3) [47]. Compared with non-MTM-HCC, vascular invasion is more frequent in MTM-HCC. While macrovascular invasion can be inferred from the imaging finding directly, the assessment of MVI and vascular encapsulated tumor clusters (VETC) remains challenging. The imaging findings associated with MVI and VETC are detailed in Supplementary Material 2.

In addition to semantic features, studies also unveiled the potential of radiomic features in diagnosing MTM-HCC. In a retrospective study of 365 HCC patients from three hospitals in China, a CT-based radiomic model was developed using 11 radiomic features extracted from AP and portal venous phase (PVP), achieving an AUC of 0.74 in the external test set [48]. In an independent cohort of advanced HCC treated with TACE and antiangiogenic therapy, patients with lower radiomics scores showed longer progression-free survival. Since histopathology is not mandatory for HCC diagnosis, the information on pathological subtype is usually unavailable in non-surgical cases. In this setting, the results of Feng and colleagues shed light on the potential of MTM and its noninvasive diagnostic models as promising decision-making tools. More recently, Li et al developed a dual-energy CT-based deep learning radiomics nomogram for predicting MTM-HCC, based on 146 patients (98% with chronic HBV infection) in China, which achieved a superior AUC compared to the conventional clinical-radiologic model in the external test set (AUC, 0.89 vs 0.79) [49]. However, the model’s performance may vary in patients with HCV infection or metabolic dysfunction-associated steatotic liver disease, and further validation is needed to assess its generalizability.

While the emerging data are promising, no model is yet sufficiently validated to be applied in clinical practice to make the noninvasive diagnosis of MTM-HCC.

#### Progenitor HCC

The available evidence indicates that progenitor HCCs tend to exhibit LR-M features, particularly the targetoid appearance, which demonstrates relatively high specificity. For example, in a single-center retrospective study, irregular tumor margin, rim APHE, lower tumor-to-liver ratio on ADC, and lower tumor-to-liver signal intensity ratio on hepatobiliary phase (HBP) were independent predictors for CK19-positive HCC, achieving a diagnostic specificity of 99.5% [31]. In addition to irregular margins and rim APHE, Chen et al reported that progenitor HCCs (either CK19 or EpCAM positive) were more prone to exhibit targetoid appearance and lack of mosaic architecture [50].

**Fig. 4:**
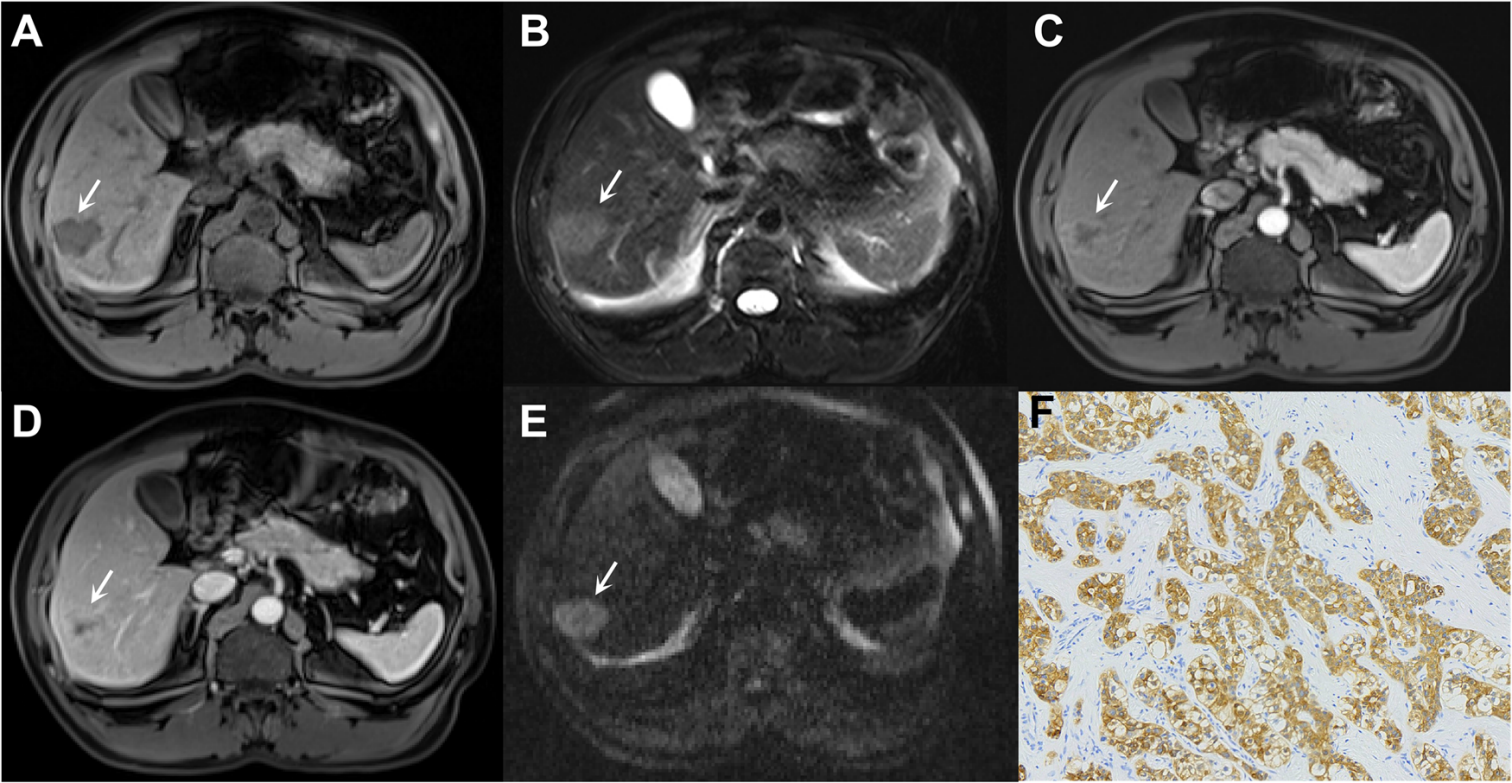
Imaging of a 48-year-old male with chronic hepatitis B virus infection and cytokeratin-19-positive HCC. The lesion (arrow, **A**–**E**) shows hypointensity on T1-weighted image (**A**), mild to moderate hyperintensity on T2-weighted image (**B**), rim hyperenhancement on arterial phase image (**C**), delayed central enhancement on PVP image (**D**), and marked diffusion restriction on diffusion weighted image (**E**). Immunohistochemistry reveals positive expression of cytokeratin-19 (**F**). The patient underwent surgical resection, with intrahepatic metastasis occurring approximately 24 months postoperatively

Furthermore, skewness on T2WI and uniformity on pre-T1WI were identified as key quantitative indicators of progenitor HCCs. Other studies identifying MRI features for predicting progenitor HCCs reached similar conclusions (Fig. 4) [51–53]. Additionally, scholars also explored radiomics-based models for diagnosing CK19-positive HCCs, which demonstrated improved predictive values over traditional radiologic models (AUCs, 0.73–0.85) [54–56].

Similar to MTM-HCC, no model is yet sufficiently validated to be applied in clinical practice to make the noninvasive diagnosis of progenitor HCC.

### Non-proliferative class

#### SH-HCC

In general, SH-HCCs have been consistently associated with the presence of intratumoral fat and smaller tumor size in published literature, although the basis for their small size has not been elucidated [41, 48, 57].

For example, in a single-center retrospective study, the SH-HCC subtype was found in 21.0% (62/295) of HCC, and fat in mass (an ancillary feature favoring HCC in LI-RADS) was more frequently detected in this subtype [41]. Furthermore, SH-HCCs were smaller and, when exceeding 10 mm in size, more frequently classified as LR-5, as confirmed in another multicenter study [57]. However, it should be noted that fat in mass is not a specific feature for SH-HCC, as it can also be observed in certain proportions of conventional, MTM, and clear-cell HCCs [58, 59]. In patients with MASH, intratumoral fat in the absence of mosaic architecture could achieve specificities reaching up to 97.6–97.7% for the diagnosis of SH-HCC [35]. Additionally, the fat component may be difficult to evaluate qualitatively when the background liver is steatotic. In this setting, quantitative MRI approaches such as proton density fat fraction (PDFF) may be helpful, although PDFF sequences tend to have low spatial resolution and so are not likely to detect small foci of fat within a tumor. The distribution of fat within a lesion (homogeneous or heterogeneous) may have diagnostic and prognostic implications, as shown by Jiang and Cannella et al in their multicenter retrospective study. The authors defined “homogenous fat” as excess intratumoral fat relative to adjacent liver without mosaic and nodule-in-nodule architecture, whereas “heterogeneous fat” referred to fat-containing tumors with either the mosaic or nodule-in-nodule architecture. Notably, HCCs with homogeneous intratumoral fat (Fleiss’ κ value, 0.38–0.41) were more frequently observed in SH-HCC and were associated with smaller tumor size in both Asian and European cohorts. Prognostically, homogeneous intratumoral fat was associated with longer recurrence-free survival and overall survival in the Asian cohort, suggesting that MRI-assessed intratumoral fat pattern should be considered as an additional factor beyond the mere presence or absence of intratumoral fat [59, 60].

**Fig. 5:**
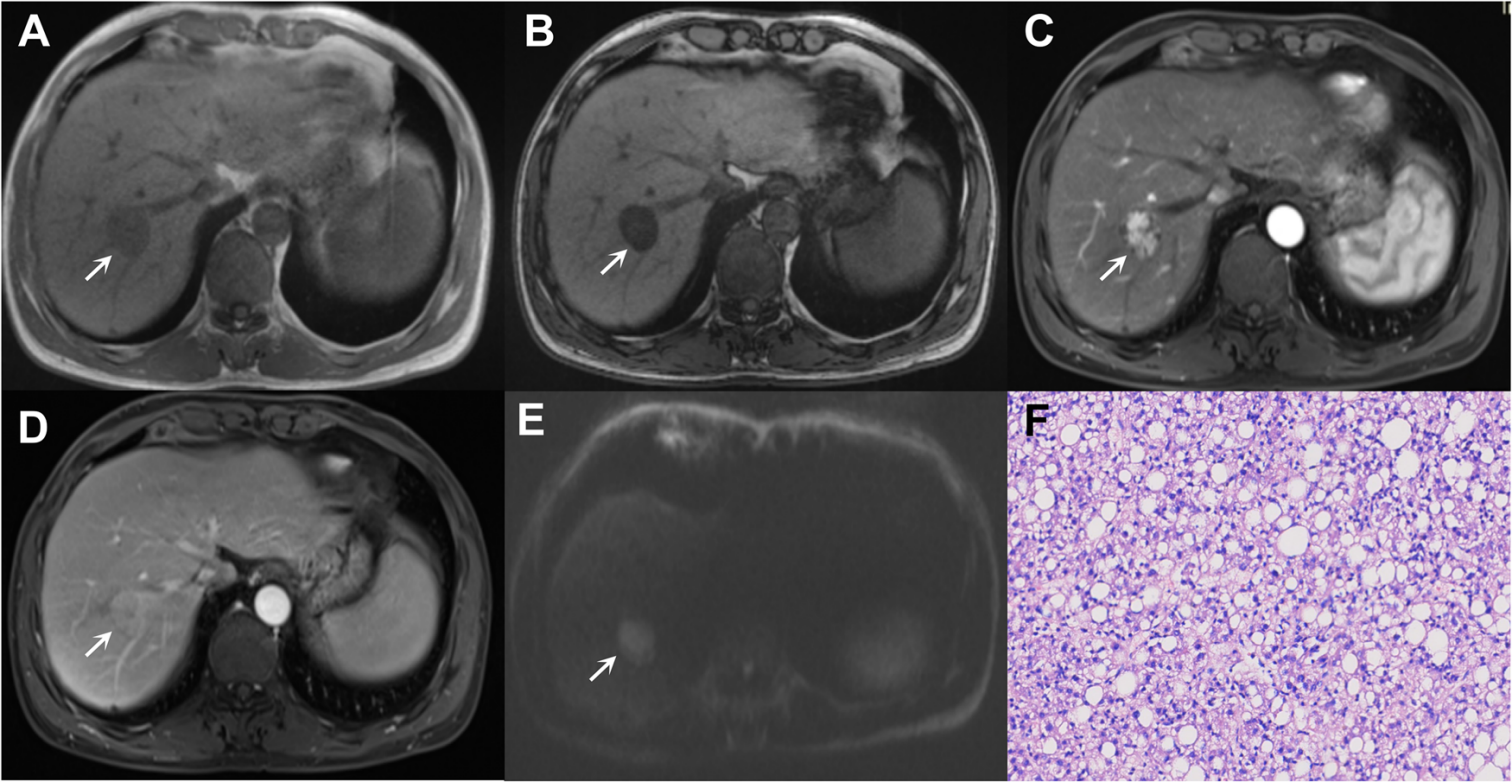
Imaging of a 52-year-old male with chronic hepatitis B virus infection and steatohepatitic HCC. The lesion (arrow, **A**–**E**) shows hypointensity on in-phase T1-weighted image (**A**) and significant signal loss on out-phase T1-weighted image (**B**), non-rim hyperenhancement on arterial phase image (**C**), no washout on PVP image (**D**), and diffusion restriction (**E**). Hematoxylin-eosin staining reveals numerous unstained fat vacuoles (**F**). The patient remains recurrence-free 30 months postoperatively

Noteworthily, unlike typical hepatocarcinogenesis in most HCCs, approximately 30% of SH-HCCs can develop directly in MASH patients without cirrhosis [61]. With metabolic dysfunction increasingly driving SH-HCC incidence, future research should identify additional radiologic hallmarks to address the shift from virus-dominated to metabolism-dominated HCC etiology (Fig. 5).

#### CTNNB1-mutated HCC

*CTNNB1* is one of the most frequently mutated genes in HCC, occurring in approximately 15–40% of cases [16, 18]. Similar to progenitor HCC, *CTNNB1*-mutated HCC is not among the eight pathological subtypes in the WHO, but still constitutes a specific genetic/molecular subtype.

**Fig. 6:**
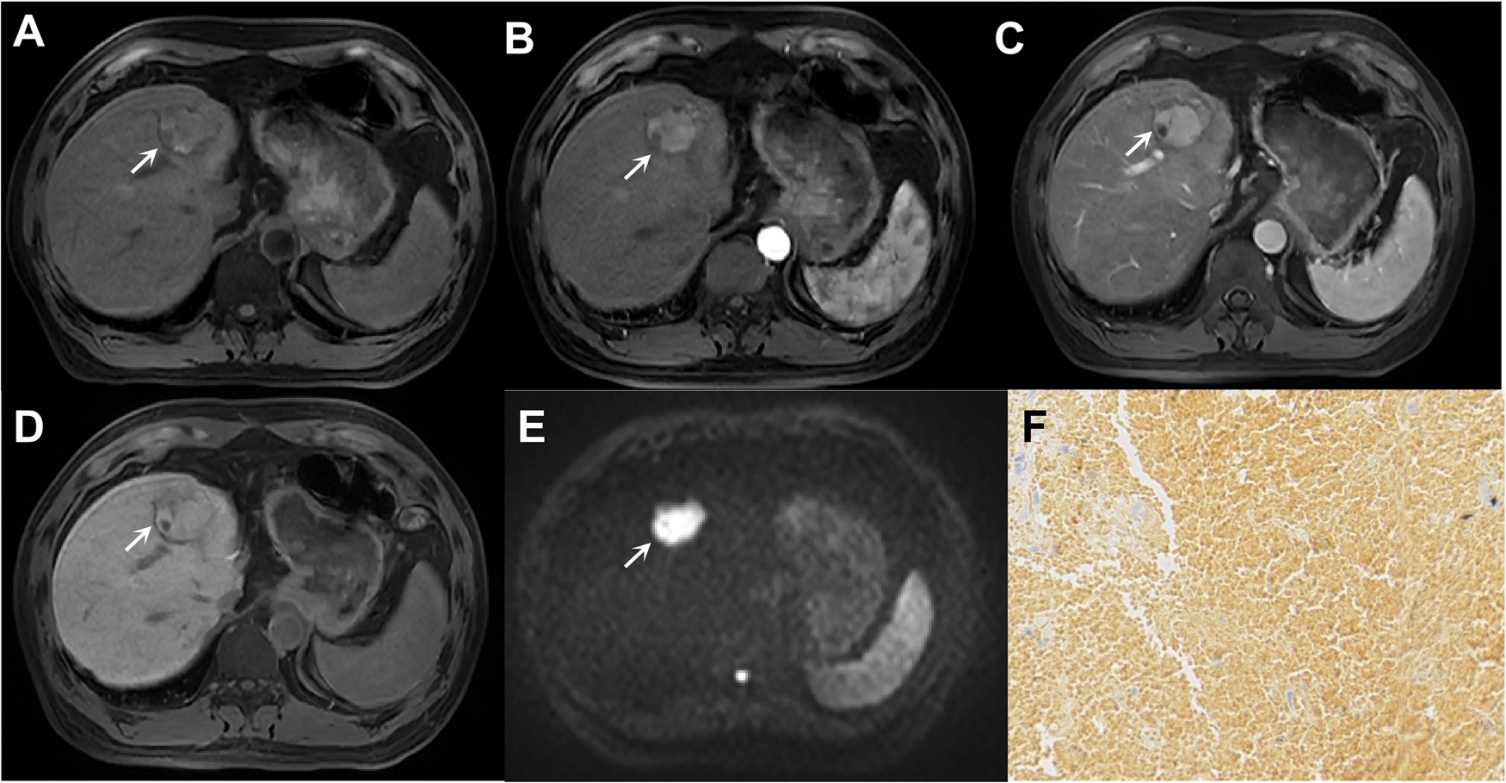
Imaging of a 56-year-old male with chronic hepatitis B virus infection and OATP-positive HCC. The lesion (arrow, **A**–**E**) shows isointensity on the T1-weighted image (**A**), non-rim hyperenhancement on the arterial phase image (**B**), and no washout on the PVP image (**C**). The lesion also demonstrates isointensity on HBP image (**D**) and marked diffusion restriction on diffusion weighted image (**E**). Immunohistochemistry reveals positive expression of OATP (**F**). The patient remains recurrence-free 32 months postoperatively

It is widely accepted that the expression of organic anion transporting polypeptide (OATP) 1B3, a transporter for hepatobiliary-specific contrast agents, decreased during hepatocarcinogenesis, resulting in hypointensity of most HCC in comparison to liver parenchyma on HBP [62, 63]. However, a small proportion ( < 15%) of HCC exhibits iso-/hyperintensity on HBP, and these HCC have been characterized by *CTNNB1* mutation (also regarded as β-catenin mutation or cholestatic-HCCs in some studies) [64]. Kitao et al retrospectively analyzed 138 resected HCCs from 121 patients and found a high enhancement ratio on HBP and a high ADC value to be more frequent in HCCs with β-catenin mutation [7]. In another study by the same team, a higher enhancement ratio on HBP was also found for β-catenin and hepatocyte nuclear factor 4α (HNF4α)-positive HCCs, implying the potential of β-catenin and HNF4α co-activation to contribute to this feature (Fig. 6) [65].

HBP-based radiomic signatures facilitate *CTNNB1*-mutated HCCs, and the incorporation of PVP features may further improve predictive performance [66, 67]. However, these studies were limited by relatively small sample sizes (< 150 cases), actors, and external validation, which may impact the generalizability and reproducibility of these models. Thus, these radiomic models are useful for showing proof of concept but are not yet sufficiently validated for clinical implementation.

### Other uncommon subtypes

Given their low incidence (< 5%), studies on uncommon HCC subtypes (e.g., scirrhous, neutrophil-rich, clear-cell, chromophobe, fibrolamellar, and lymphocyte-rich HCCs) are limited [40, 68]. Detailed findings are provided in Supplementary Material 2.

## Gaps and future directions

Despite the potential of imaging in non-invasively assessing the molecular and pathological subtypes of HCC, current studies have several key limitations.

### Gaps in the molecular and pathological classification systems

Firstly, due to the complexity and high cost of high-throughput sequencing technologies, most studies have relied on histology as a surrogate for molecular classification rather than multiomics sequencing. This is problematic because histopathologic analysis and multiomics sequencing do not demonstrate perfect correlation. Secondly, given the marked heterogeneity of HCC, it is likely that some HCCs simultaneously exhibit molecular alterations of both proliferative and non-proliferative classes (“overlap” tumors), but this possibility is ignored by current classification systems. Characterization of “overlap” HCC will require more comprehensive spatial sampling than is currently performed. Finally, because histopathologic confirmation is not mandatory to establish the definite diagnosis of HCC in at-risk patients, a large proportion of patients (particularly those with unresectable tumors) do not receive histopathologic examinations on a daily basis. Therefore, it is challenging to confirm the correlations between imaging predictors and the molecular/pathological subtypes.

### Gaps in imaging-based classification, prognostication, and prediction

First and foremost, although some studies have proposed management strategies for molecularly defined proliferative HCC or more aggressive pathological subtypes, their clinical value remains unproven [23]. On one hand, 65% of conventional HCCs and some rare subtypes lack clear prognostic and predictive significance; on the other hand, there is no consensus regarding whether imaging-classified proliferative class or aggressive pathological subtypes necessitate more aggressive intervention (e.g., surgery over ablation, wider resection margins, anatomical resections), or systemic therapies [21]. Future studies are encouraged to accumulate high-quality evidence (e.g., multi-center, large sample size, and well-standardized data collection) regarding the clinical utility of molecular and pathological classification systems. Secondly, most studies are single-center, retrospective, with relatively small sample sizes, which inevitably limits the level of evidence. Multi-center, large-cohort, and prospective studies are encouraged to fuel the clinical applicability of imaging-based prediction for molecular and pathological classification. Thirdly, the majority of the analyzed patients had chronic HBV infection, challenging the extrapolation of current findings to patients with other chronic liver disease etiologies [49, 69]. Future studies may benefit from international collaborations by including patients with more diverse etiological backgrounds. Fourthly, HCC exhibits substantial intra-individual and inter-individual heterogeneity, but pinpoint radiology–pathology spatial correlation at the voxel (radiology) and lesion-segment (pathology) level was seldom achieved in existing studies due to the retrospective nature of data collection. This makes correlating imaging findings with related molecular and pathological classification difficult. One possible way to address this challenge is to employ artificial intelligence-assisted image processing and 3D-printed patient-specific molds to achieve precise radiology–pathology correlation. This methodological innovation may help overcome current limitations and facilitate a more robust correlation between imaging features and underlying molecular or pathological profiles.

## Conclusion

Characterizing the molecular and pathological subtypes of HCC may improve individualized management and prognostication. Increasing studies have unveiled the potential of imaging as a noninvasive biomarker for molecular and pathological subtypes of HCC, with preliminary results suggesting that HCC identified as proliferative class through CT/MRI-based models demonstrate lower response rates following TACE but may potentially benefit from combined tyrosine kinase inhibitor (TKI)-ICI treatment [23, 38, 70]. Since biopsy to identify proliferative HCC is not routinely performed prior to intra-arterial or systemic treatment, suspicious imaging findings suggestive of aggressive HCC subtypes may warrant consideration of a more proactive biopsy strategy. However, the narrow representativeness of study populations and the lack of rigorous molecular-pathologic-radiologic correlation hinder the application of imaging as an effective decision-making tool. Further comprehensive and in-depth attempts are encouraging. Additionally, in light of sampling errors and the technical constraints of molecular analyses, a phenotyping system integrating clinical, imaging, pathological, and molecular phenotypes may be more attractive in modern clinical settings (e.g., unsupervised clustering analysis integrating all the above information). To further support the utility of the molecular and pathological classification of HCC, larger-scale multi-center prospective studies covering a wider range of liver diseases are highly encouraged in the future. More rigorous molecular-pathologic-radiologic correlations and integrated classification systems that take imaging features into account may provide more practical guidance for individualized management.

## Supplementary information

Supplementary information

## Abbreviations

ADC: Apparent diffusion coefficient
AFP: Alpha-fetoprotein
ALD: Alcohol-related steatotic liver disease
APHE: Arterial phase hyperenhancement
AUC: Area under the curve
CK19: Cytokeratin 19
CT: Computed tomography
DCP: Des-gamma-carboxy prothrombin
EOB-MRI: Gadoxetic acid-enhanced MRI
EpCAM: Epithelial cell adhesion molecule
HBP: Hepatobiliary phase
HBV: Hepatic B virus
HCC: Hepatocellular carcinoma
HCV: Hepatitis C virus
HNF4α: Hepatocyte nuclear factor 4α
ICI: Immune checkpoint inhibitor
LI-RADS: Liver imaging reporting and data system
MASH: Metabolically-dysfunction-associated steatohepatitis
MRI: Magnetic resonance imaging
MTM-HCC: Macrotrabecular-massive hepatocellular carcinoma
MVI: Microvascular invasion
OATP: Organic anion-transporting polypeptide
PDFF: Proton density fat fraction
PD-L1: Programmed death-ligand 1
PVP: Portal venous phase
SH-HCC: Steatohepatitic hepatocellular carcinoma
TACE: Transarterial chemoembolization
TKI: Tyrosine kinase inhibitor
VETC: Vascular encapsulated tumor clusters

## References

[1] Vogel A, Meyer T, Sapisochin G, Salem R, Saborowski A (2022) Hepatocellular carcinoma. Lancet 400:1345–1362 36084663 10.1016/S0140-6736(22)01200-4

[2] Llovet JM, Kelley RK, Villanueva A et al (2021) Hepatocellular carcinoma. Nat Rev Dis Primers 7:6 33479224 10.1038/s41572-020-00240-3 PMC13537433

[3] Singal AG, Llovet JM, Yarchoan M et al (2023) AASLD Practice Guidance on prevention, diagnosis, and treatment of hepatocellular carcinoma. Hepatology 78:1922–1965 37199193 10.1097/HEP.0000000000000466 PMC10663390

[4] Chan L-K, Tsui Y-M, Ho DW-H, Ng IO-L (2022) Cellular heterogeneity and plasticity in liver cancer. Semin Cancer Biol 82:134–149 33647386 10.1016/j.semcancer.2021.02.015

[5] Rhee H, An C, Kim H-Y, Yoo JE, Park YN, Kim M-J (2019) Hepatocellular carcinoma with irregular rim-like arterial phase hyperenhancement: more aggressive pathologic features. Liver Cancer 8:24–40 30815393 10.1159/000488540 PMC6388566

[6] Chen J, Xia C, Duan T et al (2021) Macrotrabecular-massive hepatocellular carcinoma: imaging identification and prediction based on gadoxetic acid-enhanced magnetic resonance imaging. Eur Radiol 31:7696–7704 33856520 10.1007/s00330-021-07898-7

[7] Kitao A, Matsui O, Yoneda N et al (2015) Hepatocellular carcinoma with β-catenin mutation: imaging and pathologic characteristics. Radiology 275:708–717 25668519 10.1148/radiol.14141315

[8] Taouli B, Hoshida Y, Kakite S et al (2017) Imaging-based surrogate markers of transcriptome subclasses and signatures in hepatocellular carcinoma: preliminary results. Eur Radiol 27:4472–4481 28439654 10.1007/s00330-017-4844-6 PMC5654702

[9] Calderaro J, Ziol M, Paradis V, Zucman-Rossi J (2019) Molecular and histological correlations in liver cancer. J Hepatol 71:616–630 31195064 10.1016/j.jhep.2019.06.001

[10] Rebouissou S, Nault J-C (2020) Advances in molecular classification and precision oncology in hepatocellular carcinoma. J Hepatol 72:215–229 31954487 10.1016/j.jhep.2019.08.017

[11] Boyault S, Rickman DS, De Reyniès A et al (2007) Transcriptome classification of HCC is related to gene alterations and to new therapeutic targets. Hepatology 45:42–52 17187432 10.1002/hep.21467

[12] Hoshida Y, Nijman SMB, Kobayashi M et al (2009) Integrative transcriptome analysis reveals common molecular subclasses of human hepatocellular carcinoma. Cancer Res 69:7385–7392 19723656 10.1158/0008-5472.CAN-09-1089 PMC3549578

[13] Lee J-S, Chu I-S, Heo J et al (2004) Classification and prediction of survival in hepatocellular carcinoma by gene expression profiling. Hepatology 40:667–676 15349906 10.1002/hep.20375

[14] Ally A, Balasundaram M, Carlsen R et al (2017) Comprehensive and integrative genomic characterization of hepatocellular carcinoma. Cell 169:1327–1341.e23 28622513 10.1016/j.cell.2017.05.046 PMC5680778

[15] Ziol M, Poté N, Amaddeo G et al (2018) Macrotrabecular-massive hepatocellular carcinoma: a distinctive histological subtype with clinical relevance. Hepatology 68:103–112 29281854 10.1002/hep.29762

[16] Calderaro J, Couchy G, Imbeaud S et al (2017) Histological subtypes of hepatocellular carcinoma are related to gene mutations and molecular tumour classification. J Hepatol 67:727–738 28532995 10.1016/j.jhep.2017.05.014

[17] Lachenmayer A, Alsinet C, Savic R et al (2012) Wnt-pathway activation in two molecular classes of hepatocellular carcinoma and experimental modulation by sorafenib. Clin Cancer Res 18:4997–5007 22811581 10.1158/1078-0432.CCR-11-2322 PMC3446854

[18] Zucman-Rossi J, Villanueva A, Nault J-C, Llovet JM (2015) Genetic landscape and biomarkers of hepatocellular carcinoma. Gastroenterology 149:1226–1239.e4 26099527 10.1053/j.gastro.2015.05.061

[19] Choi JH, Thung SN (2023) Advances in histological and molecular classification of hepatocellular carcinoma. Biomedicines 11:2582 37761023 10.3390/biomedicines11092582 PMC10526317

[20] Pinyol R, Sia D, Llovet JM (2019) Immune exclusion-Wnt/CTNNB1 class predicts resistance to immunotherapies in HCC. Clin Cancer Res 25:2021–2023 30617138 10.1158/1078-0432.CCR-18-3778 PMC6445700

[21] WHO Classification of Tumours Editorial Board (2019) Digestive system tumours. WHO Classification of Tumours, 5th edn. IARC, Lyon. https://publications.iarc.fr/Book-And-Report-Series/Who-Classification-Of-Tumours/Digestive-System-Tumours-2019

[22] Kim H, Jang M, Park YN (2020) Histopathological variants of hepatocellular carcinomas: an update according to the 5th edition of the WHO classification of digestive system tumors. J Liver Cancer 20:17–24 37383050 10.17998/jlc.20.1.17 PMC10035696

[23] Kang H-J, Kim H, Lee DH et al (2021) Gadoxetate-enhanced MRI features of proliferative hepatocellular carcinoma are prognostic after surgery. Radiology 300:572–582 34227881 10.1148/radiol.2021204352

[24] Kudo M (2020) Gd-EOB-DTPA-MRI could predict WNT/β-catenin mutation and resistance to immune checkpoint inhibitor therapy in hepatocellular carcinoma. Liver Cancer 9:479–490 33083276 10.1159/000509554 PMC7548850

[25] Renne SL, Woo HY, Allegra S et al (2020) Vessels encapsulating tumor clusters (VETC) is a powerful predictor of aggressive hepatocellular carcinoma. Hepatology 71:183–195 31206715 10.1002/hep.30814

[26] Llovet JM, Pinyol R, Yarchoan M et al (2024) Adjuvant and neoadjuvant immunotherapies in hepatocellular carcinoma. Nat Rev Clin Oncol 21:294–311 38424197 10.1038/s41571-024-00868-0 PMC11984461

[27] Liu L-L, Zhang S-W, Chao X et al (2021) Coexpression of CMTM6 and PD-L1 as a predictor of poor prognosis in macrotrabecular-massive hepatocellular carcinoma. Cancer Immunol Immunother 70:417–429 32770259 10.1007/s00262-020-02691-9 PMC7889680

[28] Govaere O, Komuta M, Berkers J et al (2014) Keratin 19: a key role player in the invasion of human hepatocellular carcinomas. Gut 63:674–685 23958557 10.1136/gutjnl-2012-304351 PMC3963546

[29] Kim H, Choi GH, Na DC et al (2011) Human hepatocellular carcinomas with “Stemness”-related marker expression: keratin 19 expression and a poor prognosis. Hepatology 54:1707–1717 22045674 10.1002/hep.24559

[30] Rhee H, Kim H, Park YN (2020) Clinico-radio-pathological and molecular features of hepatocellular carcinomas with keratin 19 expression. Liver Cancer 9:663–681 33442539 10.1159/000510522 PMC7768132

[31] Choi S-Y, Kim SH, Park CK et al (2018) Imaging features of gadoxetic acid-enhanced and diffusion-weighted MR imaging for identifying cytokeratin 19-positive hepatocellular carcinoma: a retrospective observational study. Radiology 286:897–908 29166246 10.1148/radiol.2017162846

[32] Guan D-X, Shi J, Zhang Y et al (2015) Sorafenib enriches epithelial cell adhesion molecule-positive tumor initiating cells and exacerbates a subtype of hepatocellular carcinoma through TSC2-AKT cascade. Hepatology 62:1791–1803 26257239 10.1002/hep.28117

[33] Salomao M, Yu WM, Brown RS, Emond JC, Lefkowitch JH (2010) Steatohepatitic hepatocellular carcinoma (SH-HCC): a distinctive histological variant of HCC in hepatitis C virus-related cirrhosis with associated NAFLD/NASH. Am J Surg Pathol 34:1630–1636 20975341 10.1097/PAS.0b013e3181f31caa

[34] Pfister D, Núñez NG, Pinyol R et al (2021) NASH limits anti-tumour surveillance in immunotherapy-treated HCC. Nature 592:450–456 33762733 10.1038/s41586-021-03362-0 PMC8046670

[35] Faure A, Dioguardi Burgio M, Cannella R et al (2024) Imaging and prognostic characterization of fat-containing hepatocellular carcinoma subtypes. Radiol Med 129:687–701 38512627 10.1007/s11547-024-01807-w

[36] Audard V, Grimber G, Elie C et al (2007) Cholestasis is a marker for hepatocellular carcinomas displaying beta-catenin mutations. J Pathol 212:345–352 17487939 10.1002/path.2169

[37] Ruiz de Galarreta M, Bresnahan E, Molina-Sánchez P et al (2019) β-Catenin activation promotes immune escape and resistance to anti-PD-1 therapy in hepatocellular carcinoma. Cancer Discov 9:1124–1141 31186238 10.1158/2159-8290.CD-19-0074 PMC6677618

[38] Bao Y, Li J-X, Zhou P et al (2023) Identifying proliferative hepatocellular carcinoma at pretreatment CT: implications for therapeutic outcomes after transarterial chemoembolization. Radiology 308:e230457 37642572 10.1148/radiol.230457

[39] Yoon JK, Choi J-Y, Rhee H, Park YN (2022) MRI features of histologic subtypes of hepatocellular carcinoma: correlation with histologic, genetic, and molecular biologic classification. Eur Radiol 32:5119–5133 35258675 10.1007/s00330-022-08643-4

[40] Bello HR, Mahdi ZK, Lui SK, Nandwana SB, Harri PA, Davarpanah AH (2022) Hepatocellular carcinoma with atypical imaging features: review of the morphologic hepatocellular carcinoma subtypes with radiology–pathology correlation. J Magn Reson Imaging 55:681–697 33682266 10.1002/jmri.27553

[41] Cannella R, Dioguardi Burgio M, Beaufrère A et al (2021) Imaging features of histological subtypes of hepatocellular carcinoma: implication for LI-RADS. JHEP Rep 3:100380 34825155 10.1016/j.jhepr.2021.100380 PMC8603197

[42] Rhee H, Cho E-S, Nahm JH et al (2021) Gadoxetic acid-enhanced MRI of macrotrabecular-massive hepatocellular carcinoma and its prognostic implications. J Hepatol 74:109–121 32818570 10.1016/j.jhep.2020.08.013

[43] Cha H, Choi J-Y, Park YN et al (2023) Comparison of imaging findings of macrotrabecular-massive hepatocellular carcinoma using CT and gadoxetic acid-enhanced MRI. Eur Radiol 33:1364–1377 35999373 10.1007/s00330-022-09105-7

[44] Mulé S, Galletto Pregliasco A, Tenenhaus A et al (2020) Multiphase liver MRI for identifying the macrotrabecular-massive subtype of hepatocellular carcinoma. Radiology 295:562–571 32228294 10.1148/radiol.2020192230

[45] Feng Z, Li H, Zhao H et al (2021) Preoperative CT for characterization of aggressive macrotrabecular-massive subtype and vessels that encapsulate tumor clusters pattern in hepatocellular carcinoma. Radiology 300:219–229 33973839 10.1148/radiol.2021203614

[46] Cheng J, Li X, Wang L et al (2024) Evaluation and prognostication of Gd-EOB-DTPA MRI and CT in patients with macrotrabecular-massive hepatocellular carcinoma. J Magn Reson Imaging 59:2071–2081 37840197 10.1002/jmri.29052

[47] Kim T-H, Woo S, Lee DH, Do RK, Chernyak V (2024) MRI imaging features for predicting macrotrabecular-massive subtype hepatocellular carcinoma: a systematic review and meta-analysis. Eur Radiol 34:6896–6907 10.1007/s00330-024-10671-1 PMC12058086 38507054

[48] Feng Z, Li H, Liu Q et al (2023) CT radiomics to predict macrotrabecular-massive subtype and immune status in hepatocellular carcinoma. Radiology 307:e221291 36511807 10.1148/radiol.221291

[49] Li M, Fan Y, You H et al (2023) Dual-energy CT deep learning radiomics to predict macrotrabecular-massive hepatocellular carcinoma. Radiology 308:e230255 37606573 10.1148/radiol.230255

[50] Chen J, Wu Z, Xia C et al (2020) Noninvasive prediction of HCC with progenitor phenotype based on gadoxetic acid-enhanced MRI. Eur Radiol 30:1232–1242 31529254 10.1007/s00330-019-06414-2

[51] Jeong HT, Kim M-J, Kim Y-E, Park YN, Choi GH, Choi JS (2012) MRI features of hepatocellular carcinoma expressing progenitor cell markers. Liver Int 32:430–440 22097930 10.1111/j.1478-3231.2011.02640.x

[52] Hu X-X, Wang W-T, Yang L et al (2019) MR features based on LI-RADS identify cytokeratin 19 status of hepatocellular carcinomas. Eur J Radiol 113:7–14 30927962 10.1016/j.ejrad.2019.01.036

[53] Lu M, Qu Q, Xu L et al (2023) Prediction for aggressiveness and postoperative recurrence of hepatocellular carcinoma using gadoxetic acid-enhanced magnetic resonance imaging. Acad Radiol 30:841–852 36577606 10.1016/j.acra.2022.12.018

[54] Wang W, Gu D, Wei J et al (2020) A radiomics-based biomarker for cytokeratin 19 status of hepatocellular carcinoma with gadoxetic acid-enhanced MRI. Eur Radiol 30:3004–3014 32002645 10.1007/s00330-019-06585-y

[55] Chen Y, Chen J, Zhang Y et al (2021) Preoperative prediction of cytokeratin 19 expression for hepatocellular carcinoma with deep learning radiomics based on gadoxetic acid-enhanced magnetic resonance imaging. J Hepatocell Carcinoma 8:795–808 34327180 10.2147/JHC.S313879 PMC8314931

[56] Wang H-Q, Yang C, Zeng M-S et al (2019) Magnetic resonance texture analysis for the identification of cytokeratin 19-positive hepatocellular carcinoma. Eur J Radiol 117:164–170 31307643 10.1016/j.ejrad.2019.06.016

[57] Mulé S, Serhal A, Pregliasco AG et al (2023) MRI features associated with HCC histologic subtypes: a western American and European bicenter study. Eur Radiol 33:1342–1352 35999375 10.1007/s00330-022-09085-8

[58] Auer TA, Halskov S, Fehrenbach U et al (2023) Gd-EOB MRI for HCC subtype differentiation in a western population according to the 5th edition of the World Health Organization classification. Eur Radiol 33:6902–6915 37115216 10.1007/s00330-023-09669-y PMC10511376

[59] Jiang H, Cannella R, Wu Z et al (2024) Prognostic implications of MRI-assessed intratumoral fat in hepatocellular carcinoma: an asian and european cohort study. Radiology 313:e233471 39499179 10.1148/radiol.233471

[60] Harmath C (2024) Hepatocellular carcinoma: the meaning of intratumoral fat patterns in different patient populations. Radiology 313:e242740 39499176 10.1148/radiol.242740

[61] Yasui K, Hashimoto E, Komorizono Y et al (2011) Characteristics of patients with nonalcoholic steatohepatitis who develop hepatocellular carcinoma. Clin Gastroenterol Hepatol 9:428–433 21320639 10.1016/j.cgh.2011.01.023

[62] Fowler KJ, Burgoyne A, Fraum T et al (2021) Pathologic, molecular, and prognostic radiologic features of hepatocellular carcinoma. Radiographics 41:1611–1631 10.1148/rg.2021210009 34597222

[63] Kitao A, Matsui O, Yoneda N et al (2011) The uptake transporter OATP8 expression decreases during multistep hepatocarcinogenesis: correlation with gadoxetic acid–enhanced MR imaging. Eur Radiol 21:2056–2066 10.1007/s00330-011-2165-8 21626360

[64] Kitao A, Matsui O, Yoneda N et al (2020) Gadoxetic acid-enhanced MR imaging for hepatocellular carcinoma: molecular and genetic bakground. Eur Radiol 30:3438–3447 32064560 10.1007/s00330-020-06687-y

[65] Kitao A, Matsui O, Yoneda N et al (2018) Gadoxetic acid-enhanced magnetic resonance imaging reflects co-activation of β-catenin and hepatocyte nuclear factor 4α in hepatocellular carcinoma. Hepatol Res 48:205–216 28488786 10.1111/hepr.12911

[66] Zeng F, Dai H, Li X et al (2022) Preoperative radiomics model using gadobenate dimeglumine-enhanced magnetic resonance imaging for predicting β-catenin mutation in patients with hepatocellular carcinoma: a retrospective study. Front Oncol 12:916126 36185240 10.3389/fonc.2022.916126 PMC9523364

[67] Arefan D, D’Ardenne NM, Iranpour N et al (2024) Quantitative radiomics and qualitative LI-RADS imaging descriptors for non-invasive assessment of β-catenin mutation status in hepatocellular carcinoma. Abdom Radiol 49:2220–2230 10.1007/s00261-024-04344-2 38782785

[68] Loy LM, Low HM, Choi J-Y, Rhee H, Wong CF, Tan CH (2022) Variant hepatocellular carcinoma subtypes according to the 2019 WHO classification: an imaging-focused review. AJR Am J Roentgenol 219:212–223 35170359 10.2214/AJR.21.26982

[69] Yan Z, Liu Z, Zhu G et al (2024) Gadoxetic acid-enhanced MRI-based radiomic models for preoperative risk prediction and prognostic assessment of proliferative HCC. Acad Radiol 32:157–169 10.1016/j.acra.2024.07.040 39181825

[70] Lu M, Yan Z, Qu Q et al (2024) Diagnostic model for proliferative HCC using LI-RADS: assessing therapeutic outcomes in hepatectomy and TKI-ICI combination. J Magn Reson Imaging 61:134–147 10.1002/jmri.29400 38647041

