# Supplementary material for: Imaging for molecular and pathological subtyping of hepatocellular carcinoma—a critical appraisal and future directions
Source: Eur Radiol. 2025 Oct 30;36(4):2744–63. doi: 10.1007/s00330-025-12075-1 (PMC13035535; doi:10.1007/s00330-025-12075-1)
Supplement: Supplementary file 1 — Supplementary information [file 330_2025_12075_MOESM1_ESM.pdf]

Imaging for molecular and pathological subtyping of hepatocellular carcinoma – A Critical Appraisal and Future Directions

ELECTRONIC SUPPLEMENTARY MATERIAL

Supplementary Material 1. Pathological characteristics of uncommon pathological subtypes.

1.1 Clear-Cell HCC has been found in 3-7% of HCC and can be identified by diffuse glycogen deposits in most tumor cells (>80%), which do not stain with hematoxylin-eosin, leaving behind the characteristic clear cytoplasm.[1] To date, knowledge regarding the clinical correlates or molecular characteristics of this subtype remains scarce, but studies have indicated that clear-cell HCC may be associated with longer overall survival compared to non-clear-cell HCCs and tends to recur later after curative-intent resection.[2]

1.2 Scirrhous HCC has been observed in ~4% of HCC and characterized by dense intratumoral fibrous stroma, occupying at least 50% of the tumor. Molecularly, *TSC1/TSC2* mutations and TGFβ (*proliferative*) pathway activation have been demonstrated in these tumors.[3] Pathologically, this subtype has been associated with increased expressions of hepatic stem cell markers such as cytokeratin 7 (CK7), CK19, or CD133 (*proliferative*).[4,5] Notably, when schirrhous HCC is located beneath the liver capsule, it often leads to capsular retraction, with abundant fibrous stroma believed to play a major role.[6,7] Clinically, the prognosis of scirrhous HCC compared to conventional HCC is variable, with studies reporting similar or worse outcomes.[4,6]

1.3 Chromophobe HCC is a rare (less than 3%) subtype of HCC and has been characterized by tumor cells with a light amphophilic to eosinophilic cytoplasm.[8] Molecularly, this subtype has been associated with the alternative lengthening of telomeres (ALT) through a telomere-independent mechanism.[9] Clinically, chromophobe HCC has been reported to have a prognosis comparable to that of conventional HCC.[10]

1.4 Fibrolamellar HCC is another rare (~1%) subtype, characterized by neoplastic hepatocytes with prominent intratumoral fibrosis.[10,11] Molecularly, the *DNAJB1-PRKACA* gene fusion has been identified in all analyzed cases of fibrolamellar HCC.[12] This phenotype typically occurs in younger and female patients without background liver diseases, with normal liver function and AFP levels.[13] It is not yet known why fibrolamellar HCC is seen predominantly in this demographic group.

***1.5 Neutrophil-rich HCC*,** also known as the granulocyte-colony stimulating factor (G-CSF) producing HCC, is a very rare subtype (<1%) characterized by diffuse neutrophilic infiltration and positive G-CSF staining.[14] Clinically, patients with neutrophil-rich HCC exhibit elevated white blood cell counts, C-reactive protein, and interleukin-6 levels and a worse prognosis in comparison to those with conventional HCC.[15]

1.6 Lymphocyte-rich HCC has been reported in <1% of HCC and characterized by abundant lymphocytic infiltrate (predominantly CD4+ and CD8+ T cells) outnumbering the tumor cells.[16,17] Lymphocyte-rich HCC has been linked to a favorable clinical outcome and shows higher programmed death-ligand 1 (PD-L1) expression than conventional HCC.[18]

Supplementary Material 2. Imaging findings associated with microvascular invasion (MVI) and vascular encapsulated tumor clusters (VETC).

MVI is a well-established prognostic factor in HCC. MVI can be diagnosed pathologically by microscopic examination of resected surgical specimens, and it is characterized by a cluster of tumor cells located in the peritumoral liver vessels. Several MRI features can predict MVI status preoperatively, including semantic characteristics (e.g., larger tumor size, tumor number, non-smooth tumor margin, rim arterial phase hyperenhancement [APHE], and peritumoral abnormalities), combinations of imaging findings (e.g., the “VICT2 trait”), and radiomics.[19,20] Lee et al. retrospectively included 197 patients with single HCC (≤5 cm) who underwent Gd-EOB-DTPA MRI.[21] They found that non-smooth tumor margin, arterial peritumoral enhancement and peritumoral hypointensity on HBP were independent predictors for MVI. The combination of all three features achieved a specificity of 99.3% for MVI prediction. Furthermore, even without HBP-MRI, the “VICT2 trait” (i.e., peritumoral portal venous phase hypoenhancement, incomplete “capsule”, corona enhancement and peritumoral mild-moderate T2 hyperintensity) derived from ECA-MRI also demonstrated comparable performance to peritumoral hypointensity on HBP in predicting MVI status and recurrence-free survival (Accuracy, 73% vs. 70%; Concordance index, 0.62 vs. 0.61).[20] However, interobserver variability of these semantic features remains moderate, particularly in smaller tumors.[22] Additionally, Xia et al. developed a CT-based radiomics model for MVI prediction by including 773 patients from four medical centers. The hybrid model, which integrated the radiomics signature with clinical-radiologic characteristics, achieved an AUC of 0.84 in the external test set.[23]

Vascular Encapsulated Tumor Clusters (VETC) represents a novel vascular pattern, first described by Fang et al. in 2015, characterized by sinusoidal blood vessels that encapsulate individual tumor clusters, forming intricate cobweb-like vascular networks.[24] This unique vascular structure facilitates the detachment and hematogenous dissemination of endothelium-coated tumor cell clusters via a mechanism independent of epithelial-mesenchymal transition (EMT). Clinically, the presence of VETC has been correlated with higher rates of postoperative recurrence, increased metastatic potential, and worse survival outcomes following surgical resection.

Recently, an increasing number of studies have been dedicated to investigate the potential of imaging to predict VETC in HCC. For example, Feng et al. conducted a multicenter retrospective study of 271 patients with HCCs, and demonstrated that tumor size >5 cm and the presence of intratumoral necrosis on CT were independent predictors of the VETC pattern.[25] Interesting, VETC pattern was more frequent in MTM-HCC rather than non-MTM-HCC.[26] Another single center study further revealed that VETC-positive HCCs exhibited a higher frequency of imaging features associated with poor prognosis, including non-smooth tumor margin, targetoid appearance, intratumoral artery, and heterogeneous enhancement with septations or irregular ring-like structure.[27] By combining the above imaging features, the CT-based model achieved similar diagnostic performance to the MRI-based model for VETC (AUC, 0.80 vs. 0.81).

The preoperative identification of VETC-positive HCCs holds significant clinical implications, potentially guiding personalized treatment strategies—such as anti-angiogenic therapy—and improving patient prognosis. Further research is warranted to refine imaging biomarkers and optimize therapeutic decision-making.

Supplementary Material 3. Imaging characteristics of uncommon pathological subtypes.

Most scirrhous HCCs exhibited delayed enhancement on contrast-enhanced CT/MRI, which was primarily attributed to the presence of abundant fibrous components. This enhancement pattern frequently resulted in the misdiagnosis of scirrhous HCCs as intrahepatic cholangiocarcinoma (ICC).[28] However, T2 central darkness, capsule and septum were more frequently observed in scirrhous HCCs, whereas targetoid appearance on DWI and HBP were more frequently observed in ICCs.[29] Compared to conventional HCCs, scirrhous HCCs are more frequently found in the subcapsular area and with an ill-defined tumor margin, rim APHE, delayed enhancement, and hepatic surface retraction.[6] Although a similar or worse prognosis has been reported for scirrhous HCC in comparison to conventional HCC, its clinical outcomes remain largely unclear due to limited publications.[10]

Additionally, the clear-cell subtype has been associated with more frequent intratumoral fat components on imaging, necessitating differentiation from SH-HCC, while the fibrolamellar subtype may mimic focal nodular hyperplasia and should be considered in young patients with large intrahepatic lesions and minimal symptoms.[30–32] Further research is required to validate these rare variants and identify their imaging features for a deeper understanding of these uncommon HCC subtypes.

References

1. Liu Z, Ma W, Li H, Li Q. Clinicopathological and prognostic features of primary clear cell carcinoma of the liver. *Hepatol Res*. 2008;38:291–299.

2. Li T, Fan J, Qin L-X, et al. Risk factors, prognosis, and management of early and late intrahepatic recurrence after resection of primary clear cell carcinoma of the liver. *Ann Surg Oncol*. 2011;18:1955–1963.

3. Calderaro J, Couchy G, Imbeaud S, et al. Histological subtypes of hepatocellular carcinoma are related to gene mutations and molecular tumour classification. *J Hepatol*. 2017;67:727–738.

4. Rhee H, An C, Kim H-Y, Yoo JE, Park YN, Kim M-J. Hepatocellular Carcinoma with Irregular Rim-Like Arterial Phase Hyperenhancement: More Aggressive Pathologic Features. *Liver Cancer*. 2019;8:24–40.

5. Renne SL, Sarcognato S, Sacchi D, et al. Hepatocellular carcinoma: a clinical and pathological overview. *Pathologica*. 2021;113:203–217.

6. Kim SH, Lim HK, Lee WJ, Choi D, Park CK. Scirrhous hepatocellular carcinoma: comparison with usual hepatocellular carcinoma based on CT-pathologic features and long-term results after curative resection. *Eur J Radiol*. 2009;69:123–130.

7. Murtha-Lemekhova A, Fuchs J, Schulz E, et al. Scirrhous Hepatocellular Carcinoma: Systematic Review and Pooled Data Analysis of Clinical, Radiological, and Histopathological Features. *J Hepatocell Carcinoma*. 2021;8:1269–1279.

8. Kang HJ, Oh J-H, Kim YW, et al. Clinicopathological and molecular characterization of chromophobe hepatocellular carcinoma. *Liver Int*. 2021;41:2499–2510.

9. Wood LD, Heaphy CM, Daniel HD-J, et al. Chromophobe hepatocellular carcinoma with abrupt anaplasia: a proposal for a new subtype of hepatocellular carcinoma with unique morphological and molecular features. *Mod Pathol*. 2013;26:1586–1593.

10. WHO Classification of Tumours Editorial Board. Digestive System Tumours. WHO Classification of Tumours. 5th Edition. 2019. https://publications.iarc.fr/Book-And-Report-Series/Who-Classification-Of-Tumours/Digestive-System-Tumours-2019

11. Calderaro J, Ziol M, Paradis V, Zucman-Rossi J. Molecular and histological correlations in liver cancer. *J Hepatol*. 2019;71:616–630.

12. Honeyman JN, Simon EP, Robine N, et al. Detection of a recurrent DNAJB1-PRKACA chimeric transcript in fibrolamellar hepatocellular carcinoma. *Science*. 2014;343:1010–1014.

13. Auer TA, Halskov S, Fehrenbach U, et al. Gd-EOB MRI for HCC subtype differentiation in a western population according to the 5th edition of the World Health Organization classification. *Eur Radiol*. 2023;33:6902–6915.

14. Nagata H, Komatsu S, Takaki W, et al. Granulocyte colony-stimulating factor-producing hepatocellular carcinoma with abrupt changes. *World J Clin Oncol*. 2016;7:380–386.

15. Aita K, Seki K. Carcinosarcoma of the liver producing granulocyte-colony stimulating factor. *Pathol Int*. 2006;56:413–419.

16. Ahn B, Ahn H-S, Shin J, et al. Characterization of lymphocyte-rich hepatocellular carcinoma and the prognostic role of tertiary lymphoid structures. *Liver Int*. 2024. doi:10.1111/liv.15865

17. Tsutsui K, Nakayama M, Ogasawara S, et al. Clinicopathological characteristics and molecular analysis of lymphocyte-rich hepatocellular carcinoma. *Hum Pathol*. 2023;141:43–53.

18. Kurebayashi Y, Ojima H, Tsujikawa H, et al. Landscape of immune microenvironment in hepatocellular carcinoma and its additional impact on histological and molecular classification. *Hepatology*. 2018;68:1025–1041.

19. Hong SB, Choi SH, Kim SY, et al. MRI Features for Predicting Microvascular Invasion of Hepatocellular Carcinoma: A Systematic Review and Meta-Analysis. *Liver Cancer*. 2021;10:94–106.

20. Jiang H, Wei H, Yang T, et al. VICT2 Trait: Prognostic Alternative to Peritumoral Hepatobiliary Phase Hypointensity in HCC. *Radiology*. 2023;307:e221835.

21. Lee S, Kim SH, Lee JE, Sinn DH, Park CK. Preoperative gadoxetic acid-enhanced MRI for predicting microvascular invasion in patients with single hepatocellular carcinoma. *J Hepatol*. 2017;67:526–534.

22. Min JH, Lee MW, Park HS, et al. Interobserver Variability and Diagnostic Performance of Gadoxetic Acid-enhanced MRI for Predicting Microvascular Invasion in Hepatocellular Carcinoma. *Radiology*. 2020;297:573–581.

23. Xia T, Zhou Z, Meng X, et al. Predicting Microvascular Invasion in Hepatocellular Carcinoma Using CT-based Radiomics Model. *Radiology*. 2023;307:e222729.

24. Fang J-H, Zhou H-C, Zhang C, et al. A novel vascular pattern promotes metastasis of hepatocellular carcinoma in an epithelial-mesenchymal transition-independent manner. *Hepatology*. 2015;62:452–465.

25. Feng Z, Li H, Zhao H, et al. Preoperative CT for Characterization of Aggressive Macrotrabecular-Massive Subtype and Vessels That Encapsulate Tumor Clusters Pattern in Hepatocellular Carcinoma. *Radiology*. 2021;300:219–229.

26. Taniai T, Shimada S, Akiyama Y, et al. Integrative transcriptome profiling elucidates molecular and immunovascular characteristics of macrotrabecular hepatocellular carcinoma. *Hepatol (baltim Md,)*. 2025. doi:10.1097/HEP.0000000000001284

27. Pan J, Huang H, Zhang S, et al. Intraindividual comparison of CT and MRI for predicting vessels encapsulating tumor clusters in hepatocellular carcinoma. *Eur Radiol*. 2024. doi:10.1007/s00330-024-10944-9

28. Jeon TY, Kim SH, Lee WJ, Lim HK. The value of gadobenate dimeglumine-enhanced hepatobiliary-phase MR imaging for the differentiation of scirrhous hepatocellular carcinoma and cholangiocarcinoma with or without hepatocellular carcinoma. *Abdom Imaging*. 2010;35:337–345.

29. Choi S-Y, Kim YK, Min JH, et al. Added value of ancillary imaging features for differentiating scirrhous hepatocellular carcinoma from intrahepatic cholangiocarcinoma on gadoxetic acid-enhanced MR imaging. *Eur Radiol*. 2018;28:2549–2560.

30. Palm V, Sheng R, Mayer P, et al. Imaging features of fibrolamellar hepatocellular carcinoma in gadoxetic acid-enhanced MRI. *Cancer Imaging*. 2018;18:9.

31. Blachar A, Federle MP, Ferris JV, et al. Radiologists' performance in the diagnosis of liver tumors with central scars by using specific CT criteria. *Radiology*. 2002;223:532–539.

32. Stipa F, Yoon SS, Liau KH, et al. Outcome of patients with fibrolamellar hepatocellular carcinoma. *Cancer*. 2006;106:1331–1338.
